# A pathway to a stronger research culture in health policy

**DOI:** 10.1186/1743-8462-4-19

**Published:** 2007-10-10

**Authors:** Jennifer Smith-Merry, James Gillespie, Stephen R Leeder

**Affiliations:** 1 School of Social and Political Studies, The University of Edinburgh, Edinburgh, UK; 2 Australian Health Policy Institute, The University of Sydney, Sydney, Australia; 3 Menzies Centre for Health Policy; School of Public Health, The University of Sydney, Sydney, Australia; 4 Menzies Centre for Health Policy; Australian Health Policy Institute, The University of Sydney, Sydney, Australia

## Abstract

**Background:**

There are currently limited pathways into a career in health policy research in Australia, due in part to a serious absence of health policy research capability in Australian universities.

**Discussion:**

We define what we consider health policy research and education should comprise. We then examine what is currently on offer and propose ways to strengthen health policy research in Australia.

**Summary:**

This paper, which is part analysis and part commentary, is offered to provoke wider debate about how health policy research can be nurtured in Australia.

## Background

A recent assessment of the capacity to conduct health services research in Australia noted that, although current workers were qualified and respected, structural limitations inhibited the discipline's influence in health system reform [1]. Health policy research is in a similar position. In their discussion of the development of health services research capacity, Pirkis et al. (2005) list among impeding factors a lack of focus amongst researchers in this field [1]. Often they must juggle other research interests and cope with insecurity of tenure resulting from short-term research contracts and dependence on grant based funding. The same situation applies to health policy research, to which we add the lack of a clear educational and career path.

In this commentary we explore the limited pathways into health policy research and how we might create new ones and strengthen those that exist. First, we examine the nature of 'health policy' teaching, drawing on major texts currently used in the teaching of policy and exploring the implications of what we find for the development of health policy as a teaching and research area. Second, we examine the ways in which health policy is currently taught at both undergraduate and postgraduate levels in Australia. Third, we explore educational paths for the development of a sustainable field of health policy researchers, and methods to locate this within Australian universities. We wish to provoke debate over the merits of such approaches to this problem and whether this will indeed assist in the development of a strengthened research culture in health policy in Australia.

## Discussion

### What is health policy?

Policy "... is the process by which governments, institutions or organisations translate their political vision into programs and actions to deliver 'outcomes' – desired changes in the real world" [2]. It can be a single statement or a set of laws, regulations, or, more vaguely, guiding principles brought to manage a particular health issue or to resolve more fundamental problems. We take health policy to be an action plan that steers the direction of a social, professional and often government response to a health related issue. The job of health policy makers is to find a way between competing needs (economic, political, value-based and so forth) in order to define compromise actions that can be taken in practice.

Consequently, 'health policy analysis' is the discovery through critical appraisal of the strengths and weaknesses of health policies or types of policy, including how they have been formulated and how they function in practice. Health policy analysis seeks to answer the questions: how can existing health policy be improved, or how can new health policy best be developed to meet social, political, economic or legal ends in relation to a health problem, taking heed of what has gone before?

### What is the nature of health policy from an academic point of view?

We define academic health policy analysis as the study of the decision-making that results in health policies, the manner in which health and health service problems are conceptualized and enter public debate, and how alternative solutions to policy problems are formulated and implemented or ignored. Recent approaches to academic health policy tend to have swung between two poles: *analysis **for **policy *and *analysis **of **policy *[3,4]. We will examine each of these in turn.

*Analysis **for **policy *focuses on the fit between a policy problem and prescription, asking the question, 'what works?' It usually assumes a rationalistic model of the policy process. Policy-making is seen as a largely technical exercise: the critical evaluation of alternative therapies, technologies, modes of organization and funding. The product of this analytical project is positive knowledge, preferably in quantitative form, to provide evidence to policy developers and decision-makers. This technical stream of policy analysis in health policy research and teaching sets out to establish quantifiable measures of the most efficient and (sometimes) most equitable ways that a society can invest in health.

The dominant academic disciplines (above all, economics) engaged in *analysis **for **policy *have concentrated on developing tools for the technical evaluation of policy options. These approaches are rational – using techniques such as cost-effectiveness analysis to quantify the effects of particular policy choices. While accepting that there is rarely one correct answer and that final decisions will often be influenced by the value positions of policymakers, *analysis **for **policy *concentrates upon linking policy decisions to clear, evidence-based criteria [5]. As Brownson et al. argue, 'when managers have complete information, they behave rationally'[6]. Policy setting institutions such as the Australian Pharmaceutical Benefits Advisory Committee (PBAC) (which determines which drugs will receive public subsidy on the basis of cost-effectiveness), are good examples of this rational model of policy analysis [7]. The PBAC applies evidence from clinical trials and cost effectiveness studies to recommend which prescribed medications should receive public funding support. The technical recommendations from the PBAC are then considered within the value-laden political process. Government decides whether to accept the PBAC recommendation. Here, therefore, politics and social values operate subsequently to the technical policy development process.

*Analysis **of **policy *by contrast recognises the messy dynamics of political and social choice and stresses that conflict and argument are fundamental elements of policy-making: 'politics is both inseparable from and preliminary to policy' [8,9]. The politicized or 'argumentative' approach gives more attention to uncovering the reasons why particular policies are adopted – often at the expense of technically more efficient alternatives. At their most extreme, these politicized approaches see choice as limited by an iron cage of institutional structures [10]. In Gill Walt's influential textbook, for example, policy *is *politics [11]. She distinguishes between the content of health policy and the processes and power relationships that create agendas and enable (or block) implementation, arguing that power and process provide the proper domain for health policy analysis. Blank and Burau's recent comparative health policy textbook also emphasises process [12]. Health policy is seen as 'those courses of action proposed or taken by a government that impact on the financing and/or provision of health services'. The core of policy analysis thus becomes the study of conflicting interests as they bear upon the process of making decisions about how to provide health services [12].

There is a danger in these accounts that health policy will become just a local instance – 'adjectival policy' [13] – of generic public policy. However, even within the mainstream of public policy, health has always been seen as posing a tougher, even intractable, set of constraints on policy makers with a complexity of a different order to education or social welfare [14]. The knowledge base – ranging from the cultural and scientific weight of medical research to the information asymmetries between providers and recipients of health care – is one thing that differentiates the nature of decision making from other areas of social policy [15].

These 'technical' and 'politicised' views of evidence and policy-making are rarely as polarized as some theories suggest. Sharp distinctions between the technical sphere of evidence and a purely political zone of decision-making risk creating an artificial analytical separation of rival black boxes of research/evidence and political decision-making [16]. From its outset the journal *Australia and New Zealand Health Policy *has recognized the need to combine both, pointing to the significance – and neglect – of the 'conflicts over values and policy choices', crucial to an understanding of policy developments in both countries [17]. Policy analysis, then, must explain how priorities are formed, and agendas filled; moving beyond both the rationalist and political frames.

Our own understanding of health policy analysis – and the technical disciplines needed for education and research in relation to it – accepts broad spectrum decision-making that includes the manner in which policies are developed, enter onto political and policy agendas and gain (or fail to achieve) sufficient political traction to be implemented. The development of skills for health policy thus requires a curriculum that enables understanding and the development of competence in relation to these policy processes.

A rounded education in health policy analysis would include a grounding in the technical disciplines of evidence assessment, including epidemiology and health economics and evaluation – the basis of most *analysis **for **policy*. But it would need to move beyond the divisions between evidence-based *analysis **for **policy *and the stress on context and decision-making processes of *analysis **of **policy*. The most articulate exponents of systematic review and other evidence-based approaches have also argued that policy-making must be seen as a drama in which language and rhetoric set the frame in which different approaches contend for power [18]. Policy analysis should also draw on political science, sociology and political economy to gain 'a rounded understanding of what it is to make and to suffer, to study and to critique, the programs and policies by which officers of the state attempt to rule'[19].

### Health policy education in Australia

We have stressed the diversity of approaches to the systematic study and research of health policy. There will always be a variety of routes, and points in individuals' careers at which a need for further training or education will become apparent. We will look in turn at several of these entry points, starting with undergraduate programs.

We systematically searched through each undergraduate health or social science course in Australia for subjects where the main component of the unit was a focus on policy making in the context of the health system. We found that of the health and social science courses offered at the 41 Australian universities 12 currently offer specific undergraduate units that focus on health policy. The health policy units that we identified are located across a broad range of courses including the Bachelor of Biomedical Sciences (Monash), Bachelor of Health Promotion (Griffith), Bachelor of Social Sciences (University of Queensland) and the Bachelor of Environmental Health (Flinders). However, the undergraduate degree in which health policy units are most commonly located is the Bachelor of Health Science, which includes a health policy unit at Deakin University, the University of Sydney, Adelaide University, Flinders and the University of Western Sydney.

None of these courses, apart from the soon to be retired Bachelor of Behavioural Health Sciences at the University of Sydney, offers more than one specifically health policy oriented unit.

Of the undergraduate units offered which specify health policy as a career path most do not teach specific health policy units. For example, the Bachelor of Health Science/Social Work at Monash University, and the Bachelor of Health Sciences degrees from Queensland University of Technology and the University of Western Australia, all list employment in health policy as a degree exit point, but do not include modules which have a primary focus on health policy [20-22]. Indeed, health policy is not offered as a major in any Australian undergraduate course. This limited undergraduate focus on health policy sets restrictions on the numbers of students able to take a health policy related honours project or a PhD project in a health policy related field and thus limits this as a path through which to develop health policy research.

While some posit that health policy should not be taught at an undergraduate level because it requires life experience and knowledge, we argue that this is not necessarily so, as demonstrated by the experience of policy teaching in similar fields. A subject area with many similarities to health policy is social policy. Social policy, though not offered as an undergraduate degree at any university in Australia, is offered as a major through various undergraduate courses, for example in the Bachelor of Social Sciences at the University of Queensland [23]. The knowledge basis gained through a major in social policy encourages students to undertake honours and from there embark on a PhD or Masters by Research in social policy. Both health policy and social policy are frequently offered as either majors or discrete programmes within undergraduate health or social sciences teaching at universities in the United Kingdom or the United States, countries that arguably have a better developed health policy research culture than Australia [24-27]. Undergraduate environmental studies programs have always integrated scientific studies with more policy focused units of study.

This deficit in undergraduate teaching of health policy is partly, but very patchily, remedied at the postgraduate level, with 21 Australian universities offering postgraduate units in health policy. Postgraduate health policy units are usually confined to individual units (or modules within survey units) within public health, public policy or (more rarely) health economics degrees. At present there are three postgraduate qualifications available in Australia (through La Trobe, Sydney and Deakin) that are entirely devoted to health policy. These are confined to the Graduate Certificate or Postgraduate Diploma level (although the University of Sydney degree will move to Master's level in 2008). The Centre for Health and Society at the University of Melbourne also offers a Masters in Social Health (Health Policy).

Most of these programs are aimed at mature age students with degrees in other disciplines, who have been working in policy positions in government, or clinicians seeking policy training. All of these postgraduate programmes offer a site for attracting students into a career in health policy research and more needs to be done in order to develop research in these programmes and encourage the course participants to enter into research higher degrees in health policy.

Another area where health policy is included but underdeveloped at a postgraduate level is in Masters of Public Health programmes, which are now taught in 18 Australian universities. While some universities offer a small taste of policy analysis in introductory core units, in most public health programs policy suffers in an already crowded program.

What of funding for health policy courses at a postgraduate level? The Commonwealth government's Public Health Education and Research Program (PHERP) has made contributions to policy a requirement for gaining its support. The most recent evaluation of PHERP listed 'policy development and review' as one of the areas where interdisciplinary teaching and research should be fostered. It offers as a model the development of Health Impact Assessment (HIA) techniques [28]. HIAs offer a valuable method for putting health concerns on public agendas, but as a model for policy education and research they remain firmly in the *analysis **for **policy *category. The introduction and expansion of units such as these within postgraduate degree programmes is a good start in developing a comprehensive understanding of health policy at a postgraduate level. We argue that these should evolve to include health policy units that focus on both *analysis **for **policy *and *analysis **of **policy *approaches to health policy. This would provide health policy competence which could be built upon through a PhD. As with our discussion of undergraduate health policy teaching above, it is important that health policy teaching at a postgraduate level not be seen as a preserve of the health sciences, but included in postgraduate suites across the social sciences.

The marginal status of health policy at both an undergraduate and postgraduate level limits students' exposure to the range of theories and methods necessary for serious research. A greater emphasis needs to be placed on health policy teaching at all levels in order to attract students into the field.

### Pathways to a stronger research culture for health policy

Currently there are many entry points to health policy research. For example, in the health policy research centre where we are based, two academics completed degrees in medicine, one has a degree in physiotherapy and two others started with undergraduate degrees in history before moving into health policy research. It is important to note that the many different pathways that currently lead to academic research in health policy are not in and of themselves a negative. On the contrary, the diversity of backgrounds of current researchers allows for a wide variety of influences to enrich health policy research. The current circuitous approach does however leave the development of health policy researchers to chance rather than design, meaning that there is no avenue in Australia for assuring that there are adequate numbers of researchers emerging in this field. For the remainder of this paper we explore pathways for developing health policy researchers which are the most direct, yet currently the most underdeveloped. We highlight three critical steps along the most direct pathway – the health policy 'streamed' undergraduate and honours student, the health policy PhD and the health policy postdoctoral fellowship and discuss other alternative opportunities for the development of health policy research.

### Undergraduate pathways

As discussed, an area which currently has little focus on health policy is undergraduate teaching. We feel that it would be appropriate to introduce a specific health policy teaching stream at this level in order to promote interest in health policy as a research area for students who want to progress toward a research career.

The lack of undergraduate training in health policy has a significant effect on the development of a research culture in health policy. In the absence of a clear educational path few emerging academics can consider this a disciplinary focus for their research. It is thus important that health policy be developed as a discrete field of study within undergraduate programmes across the health and social sciences in order to lead a pool of potential researchers toward research in health policy. While this is not the only way to engage with potential health policy research workers, we see it as an obvious one which is currently under-developed.

As discussed above our search through the current health policy offerings in Australia identified Bachelor of Health Sciences degrees as the most common site for health policy teaching to be located. The inclusion of a health policy stream or major within a health sciences degree makes some sense because those who enter into this degree have a general, as yet undirected interest in the health system which could be channelled into health policy research. Even if a student were not to go into a research career in health policy, engagement with health policy at an undergraduate level should provide them with a general introduction as to how policy decisions are made within the health system. This is especially important for students who will follow a career as a health care practitioner as it may help develop a holistic understanding of the health system. However this is not the only productive place in which to develop health policy research at an undergraduate level and health policy research as a field could be more broadly developed were it to be encouraged as an undergraduate field of study within social and political sciences. Teaching health policy in this context would enhance the *analysis **of **policy *approach, which is currently underdeveloped in health policy research [4].

Another possibility is the inclusion of health policy as a field of study within dual degrees. Health policy could be offered as a module in a dual degree which offers both a health and a social science focus. The interdisciplinary approach offered by dual degrees such as these would bring together the elements of sociology and political science with an understanding of the workings of the health care system, thereby integrating both the *analysis **for **policy *and *analysis **of **policy *approaches to health policy.

Undergraduate programme options would offer students of health policy an experience from which they could acquire the foundational knowledge and skill to take into a health policy themed honours research project. Honours is a significant year for developing research workers as it allows for the creation of an independent research project through which students are able to learn and apply a set of research skills. It gives them a taste of what research in health policy may entail and enables academics to identify and encourage the next generation of health policy researchers. We suggest the attributes listed in table 1, below, as appropriate for an honours student graduating with competency in health policy:

**Table 1:** Graduate Attributes for Honours Students Graduating with Competency in Health Policy.


*Knowledge of policy environments*, including:
◸ understanding of the Australian health system, including knowledge of both Federal and State/Territory systems
◸ a basic understanding of comparative international health policy
*Knowledge of information and evidence*, including:
◸ the ability to identify and effectively use appropriate research to understand and analyse health policy
◸ skills in evidence acquisition and analysis
◸ a basic and technical knowledge in health services evaluation
◸ a capacity to integrate qualitative and quantitative skills and information
◸ knowledge and understanding of the social and political determinants of health and health policy
◸ an understanding of the basic principles of health economics
*Knowledge of policy process*, including:
◸ knowledge of both micro and macro level views of policy
◸ knowledge of the legal, value, political and ethical influences on policy
◸ awareness of the role of leadership in policy development and implementation
*Knowledge of the didactic analysis of policy*, including:
◸ the ability to examine the congruence between values and intentions and actual policy
◸ the ability to understand and analyse aspirational policy and the importance of constructive new ideas
◸ practical knowledge of examples of policy failures and "real" policy
*Skills for effective engagement of policy stakeholders*, including:
◸ knowledge and skills in advocacy and consultation
◸ the ability to appreciate the value and ethical aspects that influence stakeholder views and positions
◸ knowledge of the breadth of policy stakeholders and the diversity of their interests and influence

### Honours to PhD

A degree with first class honours or honours 2a in health policy will in most cases ensure that students are able to conceptualise and carry out a supervised research project at doctoral level. A solid background at undergraduate and honours levels will make the transition to PhD more attractive as well as easier for the student. The benefits of a PhD are clear – the student can complete a research project that they have conceived, develop appropriate methodological and theoretical frameworks for analysis, and present findings in a manner acceptable to academic peers within a context of supervised learning. The mentoring of a good supervisor is integral to this process.

At present, there is little opportunity for students to engage with health policy through teaching at an undergraduate level and an honours project. It is vital, at the moment, therefore, that health policy academics actively seek to recruit the next generation of research workers from other places. Potential sites for recruitment include health science, political science and social science honours, masters and professional doctorate programmes, where students have developed an interest in health policy and are looking to pursue this interest further. Another recruiting tactic would be to approach individuals in career streams that might feed into masters and postgraduate programmes in health policy, and from this to a PhD in health policy. Internships in health policy research centres could be offered, and visits arranged by health policy academics to postgraduate programmes. Active collaboration between health policy academics, public servants and practitioners would also be useful.

### Postdoctoral research

Whatever tangled routes lead developing research workers toward a health policy research focus, a relevant and well structured postdoctoral experience is necessary to consolidate postgraduate training. It has recently been argued that expanded postdoctoral research fellowship programs are essential if we are to replace the estimated third to half of all Australian academics reaching retirement age over the next decade [29]. Postdoctoral research fellowships provide the best means for mentoring younger researchers in a discipline [29].

In order for a postdoctoral experience to be as productive as possible it should include a mix of autonomous and team based research and be based around a strong mentoring relationship with an academic established in health policy research. A survey of 7,600 US postdoctoral researchers conducted in 2005 found that researchers were "... more likely to be happy with their jobs and to publish copiously when they receive a lot of structured training and advice from mentors" [30]. A drawback of the long period of mentoring involved in a postdoctoral position could be that researchers are less willing to enter onto this path because of the limited salaries available compared with what they might receive in industry. Postdoctoral fellowships are funded low on the academic salary scale, at either level A or B. It is important that postdoctoral fellowship salaries allow for a comfortable standard of living, especially as most postdoctoral researchers will be at a stage in their lives where they have significant income needs such as a young family or mortgage repayments.

It is important that following on from the postdoctoral research experience, opportunities are available for researchers in health policy to obtain permanent teaching and research positions or long-term senior fellowships. The current fellowship and grant based funding available to those finishing a postdoctoral position is usually available for a maximum of three years. This leads to a lack of job security for emerging research workers [31].

## Summary

This paper has sought to define the field of health policy education and associated research and outline educational paths that can be developed in order to strengthen health policy as a research field. We invite comment on these ideas and encourage academics across Australia to find a place for health policy in their undergraduate and postgraduate curricula and to be innovative in their recruitment, development and mentoring of future health policy research workers.

## Competing interests

All authors currently work in health policy research and teaching. JSM was recently in receipt of an NHMRC Capacity Building Postdoctoral Fellowship in Health Policy.

## Authors' contributions

JSM conceived of the theme for this article and completed the research relating to the current teaching of health policy, devised the pathway and prepared the manuscript for publication. JG assisted in the development of the ideas expressed in the article, wrote the section defining health policy and assisted in the preparation of the manuscript draft. SL assisted in the development of the ideas expressed in the article and helped to prepare the manuscript draft. All authors read and approved the final manuscript.
